# Point-of-care ultrasound (POCUS) as a key tool in the diagnosis of pheochromocytoma: a case report in an elderly patient with extreme blood pressure variability

**DOI:** 10.3389/fmed.2025.1525893

**Published:** 2025-07-30

**Authors:** H. A. Nati-Castillo, Jhan S. Saavedra T., Luis Alfonso Valderrama, Esteban Ortiz-Prado, Juan S. Izquierdo-Condoy

**Affiliations:** ^1^Grupo Interinstitucional Medicina Interna (GIMI 1), Departamento Medicina Interna, Universidad Libre, Cali, Colombia; ^2^Medicina Familiar, Pontificia Universidad Javeriana, Cali, Colombia; ^3^Departamento de Nefrología, Clínica Imbanaco de Cali, Colombia; ^4^One Health Research Group, Universidad de las Américas, Quito, Ecuador

**Keywords:** point-of-care ultrasound, pheochromocytoma, early diagnosis, low- and middle-income countries, hemodynamic instability

## Abstract

**Background:**

Pheochromocytoma is a rare, life-threatening, neuroendocrine tumor originating from catecholamine-secreting chromaffin cells, with an incidence of up to 8 per million people globally each year. It manifests a broad spectrum of symptoms due to excessive catecholamine secretion, often mimicking other conditions and complicating its diagnosis. Its clinical variability poses a significant diagnostic challenge, delaying appropriate interventions–particularly in resource-limited settings.

**Case presentation:**

We report the case of a 73-years-old male who arrived at the emergency department exhibiting chest pain, palpitations, marked blood pressure fluctuations without clinical signs of tachycardia, and excessive sweating, initially suggesting a potential coronary event. Initial cardiac evaluations, including coronary angiography, were inconclusive. The application of readily available Point-of-Care Ultrasound (POCUS) provided crucial initial insights, prompting further abdominal imaging. This imaging revealed a left adrenal mass indicative of pheochromocytoma, which was subsequently confirmed through computed tomography (CT) scanning. The patient underwent urgent adrenalectomy, resulting in the stabilization of his symptoms and blood pressure levels. Histopathological analysis confirmed the diagnosis.

**Conclusion:**

This case underscores the critical role of POCUS in the emergency setting, where hypotension and hypertension may signal a high-risk scenario requiring urgent diagnosis. POCUS can significantly enhance diagnostic accuracy and influence patient outcomes. Its use can expedite the identification of pheochromocytoma and improve management strategies, particularly in settings with limited access to advanced imaging.

## 1 Introduction

Pheochromocytoma, a rare neuroendocrine tumor that arises from catecholamine-secreting chromaffin cells of neural crest origin, has an estimated incidence of 2 to 8 cases per million people annually (1, 2). However, this rarity may underestimate its true prevalence, as diagnostic oversight often leads to cases being diagnosed only posthumously (3). The tumor typically presents between the ages of 40 and 60, without a discernible bias toward any gender, and primarily presents with arterial hypertension in approximately 90% of affected individuals (4), although it accounts for only 0.2% of all hypertension diagnoses (5). In addition, patients may experience non-specific symptoms, including headaches, palpitations, sweating, and facial flushing (4). The condition can arise sporadically or be associated with hereditary syndromes, with certain studies revealing germline mutation rates among patients as high as 24%–27%, highlighting its genetic significance (6).

Diagnosing pheochromocytoma involves a combination of clinical evaluation, biochemical tests for fractionated catecholamines and metanephrines in either 24-h urine samples or plasma, and imaging techniques such as computed tomography (CT) or magnetic resonance imaging (MRI) particularly in cases of suspected metastasis or requiring detailed anatomical assessment (7, 8).

In recent years, point-of-care ultrasound (POCUS) has emerged as a valuable diagnostic tool at the bedside, offering rapid, cost-efficient evaluations while potentially reducing the need for more elaborate imaging procedures. This attribute is particularly advantageous for bridging healthcare gaps in low-resource, rural, or remote locations. Despite its extensive application in the developed contexts of North America and Europe, the availability of POCUS within healthcare settings across Latin America is notably scarce (9, 10).

This report details the case of an elderly patient with multiple comorbidities and marked blood pressure variability, who was subjected to diagnostic ambiguities and critical health threats. The application of POCUS within the emergency department facilitated a timely and accurate diagnosis, significantly altering the patient’s management and outcome.

## 2 Case presentation

A 73-years-old male patient with a history of multiple comorbidities, including arterial hypertension, type 2 diabetes mellitus not requiring insulin, liver cirrhosis, portal hypertension, obstructive sleep apnea-hypopnea syndrome, benign prostatic hyperplasia, hypothyroidism, and a prior episode of acute myocardial infarction without obstructive coronary lesions (MINOCA), with a left ventricular ejection fraction (LVEF) of 65% 1 year earlier. At initial presentation, he arrived at the emergency department due to chest pain at rest, without radiation. Non-ST-segment elevation acute coronary syndrome (NSTE-ACS) was considered. An invasive coronary angiography was performed, revealing normal epicardial coronary arteries with slow intracoronary flow. Both MINOCA and Takotsubo syndrome (TTS) were considered. Transthoracic echocardiography revealed an LVEF of 37% with extensive segmental wall motion abnormalities. Cardiac magnetic resonance imaging did not demonstrate cardiomyopathy, and the patient was discharged after symptom resolution.

Seven days later, the patient returned to the emergency department with oppressive chest pain at rest, accompanied by a generalized headache and nausea. On physical examination, blood pressure was markedly elevated. After cardiology consultation, antihypertensive and anti-anginal therapy was adjusted with the addition of trimetazidine, resulting in transient blood pressure stabilization.

**FIGURE 1:**
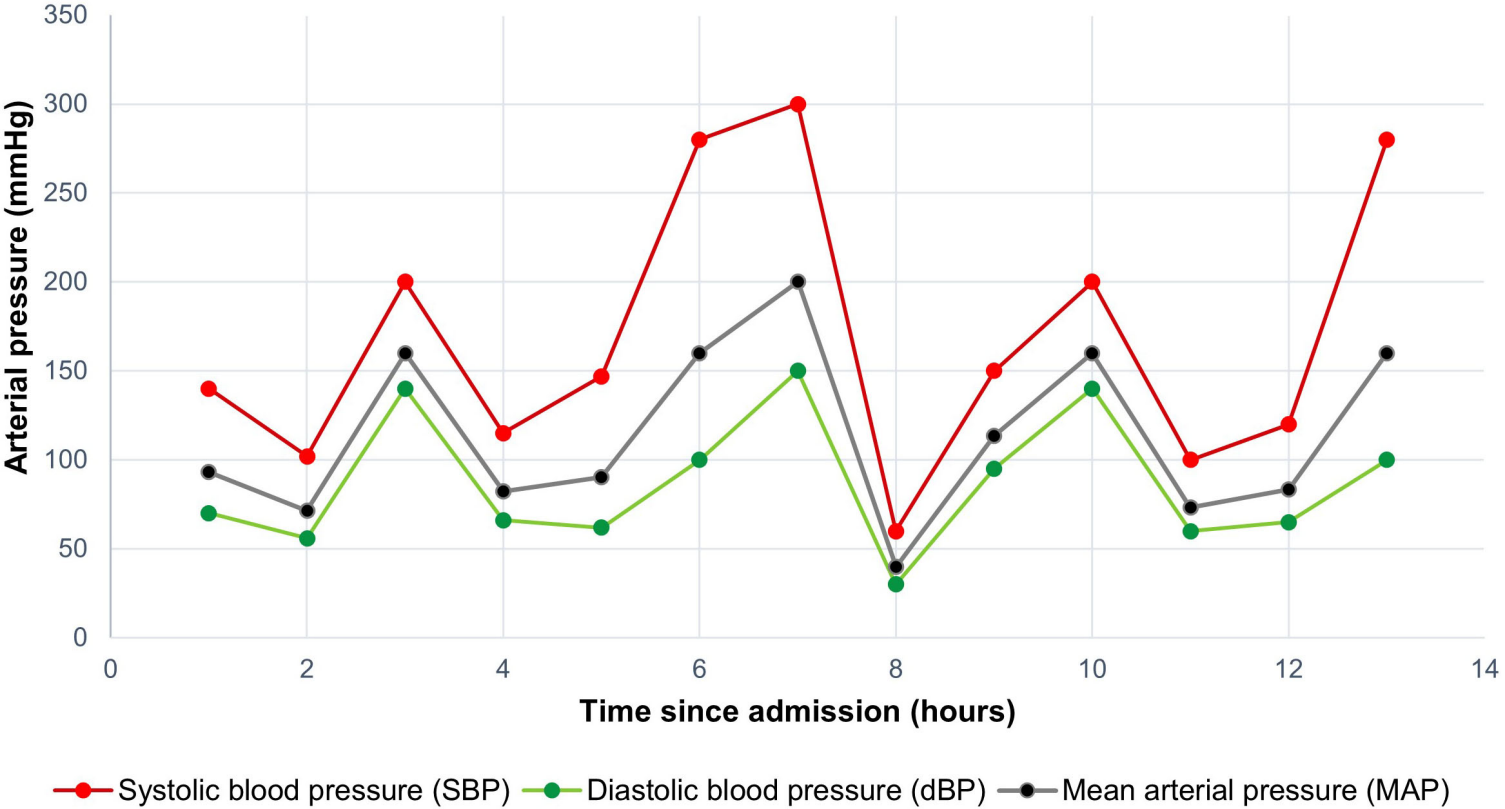
Blood pressure variability of the patient within the first 14 h of care in the emergency room.

Subsequently, marked fluctuations in blood pressure were observed, with episodes of severe hypotension [diastolic blood pressure (DBP) as low as 40 mmHg] alternating with hypertensive crises (DBP up to 200 mmHg) (Figure 1), while heart rate remained within normal limits. Blood pressure measurements in all four extremities revealed significant asymmetry in the left lower limb. Based on these findings, acute aortic syndrome was suspected, prompting a thoracic aortic angiotomography, which showed normal results. Given the persistence of extreme blood pressure variability in the emergency setting, POCUS was performed. This revealed a well-defined, rounded lesion at the level of the left renal pole, raising the suspicion of secondary arterial hypertension due to an underlying mass. In this context, pheochromocytoma was suspected, and 24-h urinary catecholamines and metanephrines were ordered. In light of these findings, pheochromocytoma was strongly suspected, and 24-h urinary catecholamines and metanephrines were requested. However, given the hemodynamic instability, the patient was transferred to the intensive care unit (ICU) for close monitoring.

**FIGURE 2:**
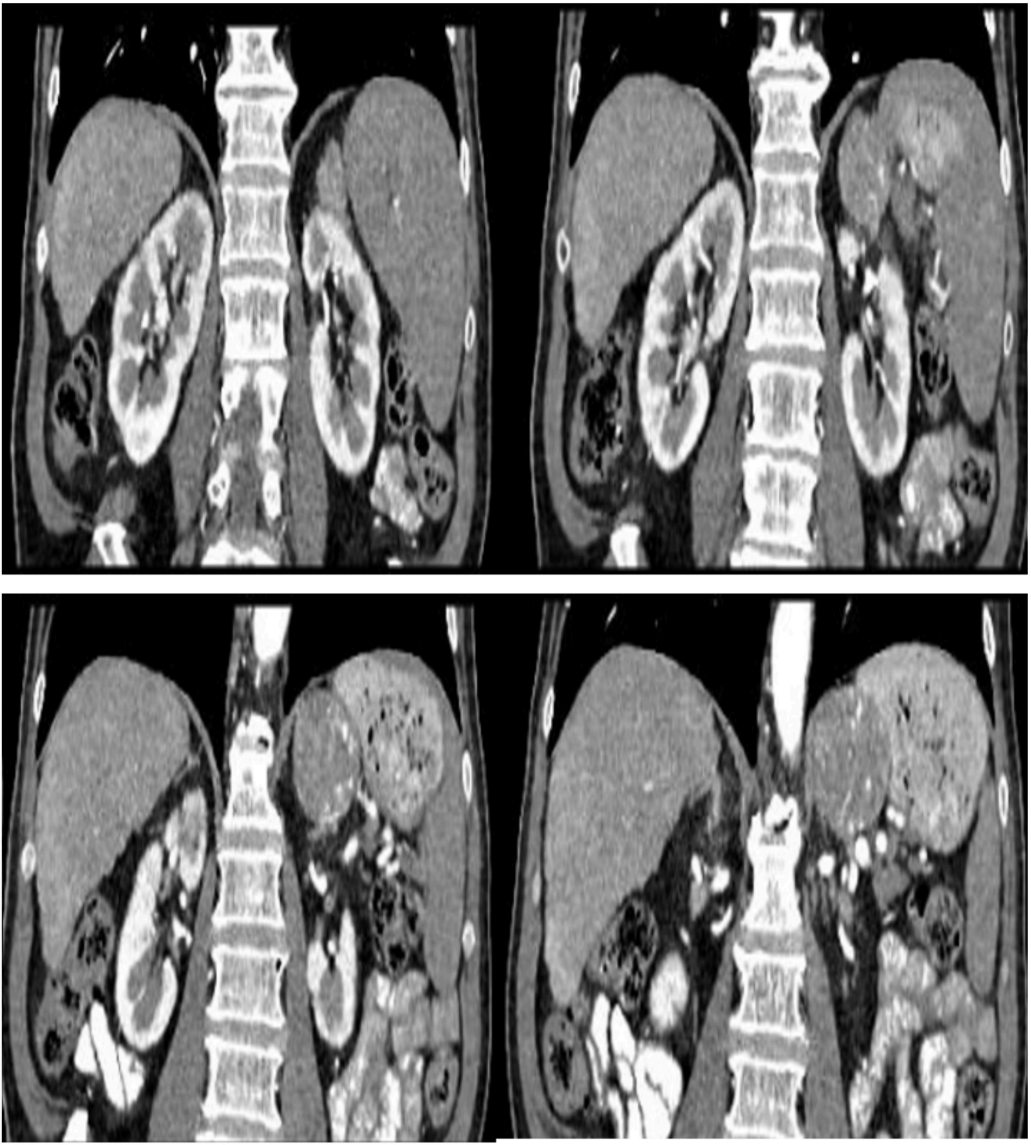
Contrast abdominal-pelvic computed tomography. Left adrenal gland with solid lesion with heterogeneous contrast uptake, with central and peripheral penetrating vessels, size approximately 4.8 cm in diameter. No fatty content or calcifications were observed in its interior.

During ICU stance, an abdominopelvic CT scan with oral and intravenous contrast was performed. Imaging revealed a solid left adrenal mass measuring approximately 4.8 cm, with heterogeneous contrast enhancement, internal vascularization, and no fat or calcifications (Figure 2). The right adrenal gland appeared normal. Additional findings included signs of chronic liver disease with portal hypertension and collateral circulation in the splanchnic bed. Eighteen hours after the initial presentation and considering the imaging findings, persistent symptoms, and high risk of complications, emergency adrenalectomy was indicated. During surgery, the tumor–approximately 5 cm in diameter–was visualized in the left adrenal gland, located between the spleen, stomach, and left kidney, inferior to the tail of the pancreas. Throughout the procedure, the patient exhibited significant blood pressure fluctuations.

**FIGURE 3:**
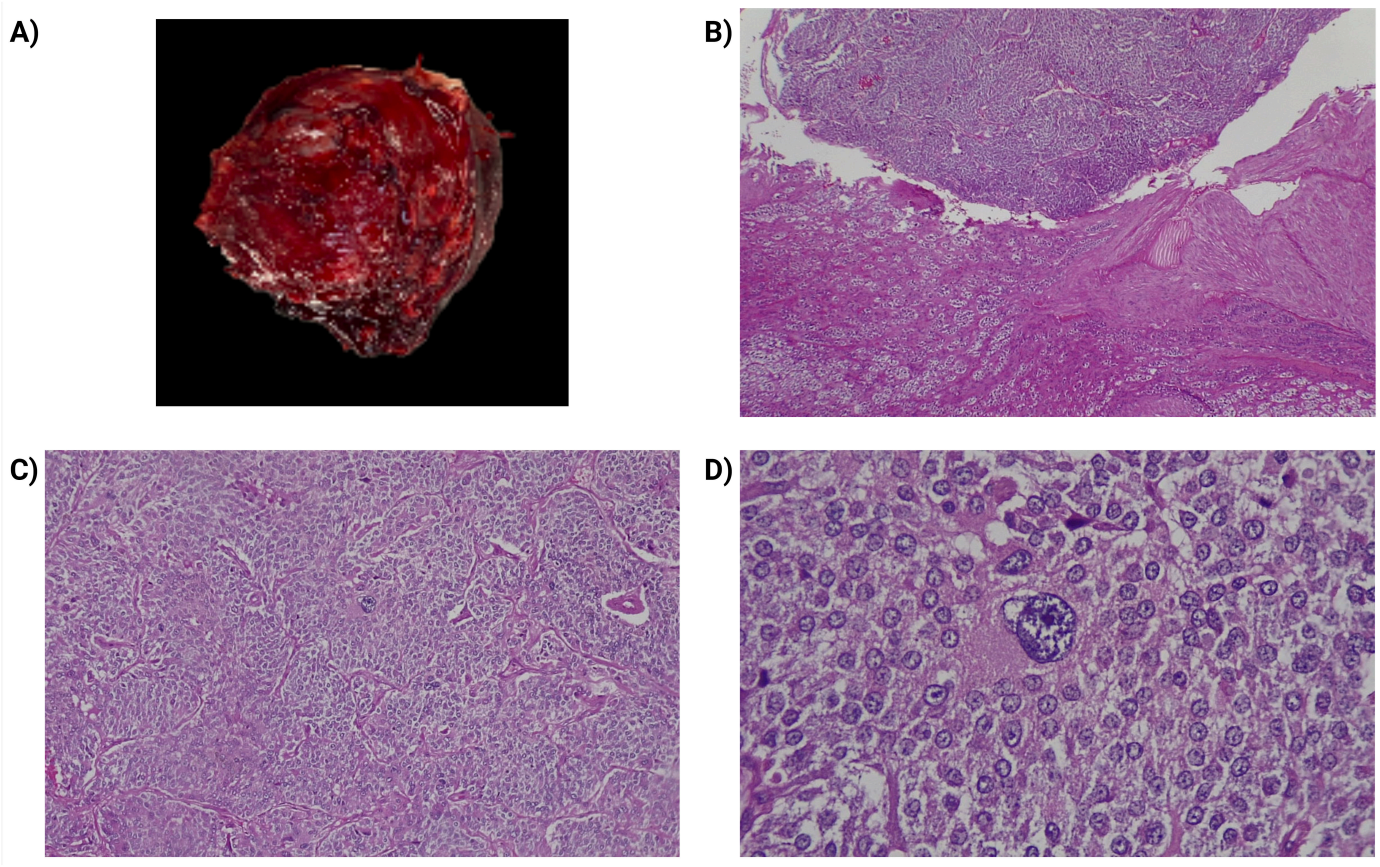
Pathological study of the patient’s lesion. **(A)** Macroscopic image of the lesion revealed a weight of 85 grams, with a size: 4.7 × 4.7 × 4.5 cm, with the appearance of a solid yellowish-brown internal surface, with violaceous areas, well delimited by a thin external capsule. **(B)** Transition between the adrenal cortex of usual histology (lower part of the image) and the tumor (upper part). With hematoxylin and eosin (H&E), 100×. **(C)** Pheochromocytoma, cells arranged in a nested pattern (zellballen) and trabeculae (H&E, 200×). **(D)** Pheochromocytoma, the cells of the lesion are large, polygonal, and demonstrate fine, granular cytoplasm. Presence of pleomorphism, (H&E, 400×).

At the conclusion of surgery, the patient achieved hemodynamic stability. However, postoperative vasopressor support was required for 12 h, and vasoactive agents were weaned over the subsequent 24 h, with favorable clinical evolution and no recurrence of symptoms. Forty-eight hours later, the patient remained clinically stable, and hospital discharge was indicated. Post-discharge, histopathological examination confirmed the diagnosis of pheochromocytoma. According to the Pheochromocytoma of the Adrenal Gland Scoring Scale (PASS), the lesion scored 3 points, consistent with a benign pheochromocytoma (Figure 3).

## 3 Discussion

We present a case characterized by diagnostic uncertainty due to an unusual pattern of periodic, severe, and sustained episodes of hypertension and hypotension triggered by pheochromocytoma. Although the exact mechanism underlying these blood pressure fluctuations remains unclear, some studies have suggested they may result from sympathetic or central nervous system ischemic reflexes (11, 12). Notably, despite significant blood pressure variability, the patient did not develop acute myocardial injury, which is often observed in cases of catecholamine-induced cardiomyopathy (13, 14).

Although biochemical confirmation remains the standard first step in diagnosing pheochromocytoma–particularly in accordance with Endocrine Society guidelines (7), an expedited diagnostic and therapeutic approach was deemed necessary in this case. The patient experienced recurrent hypertensive crises and episodes of profound hypotension, posing an imminent threat of cardiovascular or cerebrovascular complications. Accordingly, clinical judgment, supported by multidisciplinary consensus and the family’s informed consent, prioritized immediate intervention over strict adherence to diagnostic timelines.

In the context of high-risk endocrine emergencies, the literature supports context-sensitive, adaptive decision-making. In such scenarios, experienced clinical reasoning may ethically justify deviations from standard protocols when patient safety is at stake (15–17). Three key deviations from standard care in this case require clarification: First, emergent adrenalectomy was performed without awaiting biochemical confirmation. The decision was based on the patient’s life-threatening hemodynamic instability and the detection of a hypervascular adrenal mass on both POCUS and CT. No alternative diagnosis adequately explained the patient’s presentation, and delaying surgery would have likely increased the risk of cardiovascular collapse. Thus, immediate surgical intervention was clinically and ethically justified. Second, an initial thoracic CT angiography was prioritized over complete aortic imaging. This decision was guided by strong clinical suspicion of acute thoracic aortic pathology and the limited imaging availability at the time. While a more extensive aortic study may have expedited diagnosis in retrospect, the initial imaging approach reflected the urgency and resource constraints faced during the acute presentation. Finally preoperative alpha-adrenergic blockade was omitted. The patient’s hemodynamic instability, liver dysfunction, and portal hypertension contraindicated pharmacologic preparation. This decision was reached via interdisciplinary consensus and family approval, consistent with ethical principles in emergency care and prior reports of similar management strategies in life-threatening pheochromocytoma crises (15–17).

Adrenalectomy was performed prior to receiving the urinary metanephrine results due to the patient’s unstable condition and the high likelihood of a functional adrenal tumor based on imaging and clinical presentation. In resource-limited settings, such adaptive decision-making may be ethically and clinically justified, particularly when delays could be life-threatening. This approach aligns with published literature describing similar pheochromocytoma crises managed with early surgical intervention despite incomplete biochemical confirmation (15, 16, 18).

Bekelaar et al. (15), for instance, described a patient in cardiogenic shock with blood pressure fluctuations ranging from 45 to 290 mmHg who stabilized rapidly following emergency adrenalectomy (15). Similarly, Kakoki et al. (16) reported successful surgical treatment of a multisystem pheochromocytoma crisis without confirmed catecholamine levels (16). These cases reinforce the role of clinical acumen and radiologic evidence in justifying early surgery in extreme scenarios where hemodynamic instability may preclude standard workup. While incidental non-functional adrenal adenomas are relatively common in older adults, the imaging features in this case–including size > 4 cm, intense heterogeneous contrast enhancement, internal vascularity, and absence of fat–are atypical for incidentalomas and instead raised strong suspicion for a functional adrenal tumor, consistent with established radiological criteria for pheochromocytoma.

Ethically, this decision is supported by the principles outlined in the Code of Ethics for Emergency Physicians, which prioritize patient welfare over protocol adherence in life-threatening contexts. In this case, the strategy respected the principles of beneficence, non-maleficence, and autonomy, given the interdisciplinary agreement and the family’s consent. Emergency medicine frequently requires such flexibility, particularly in under-resourced environments (19).

**FIGURE 4:**
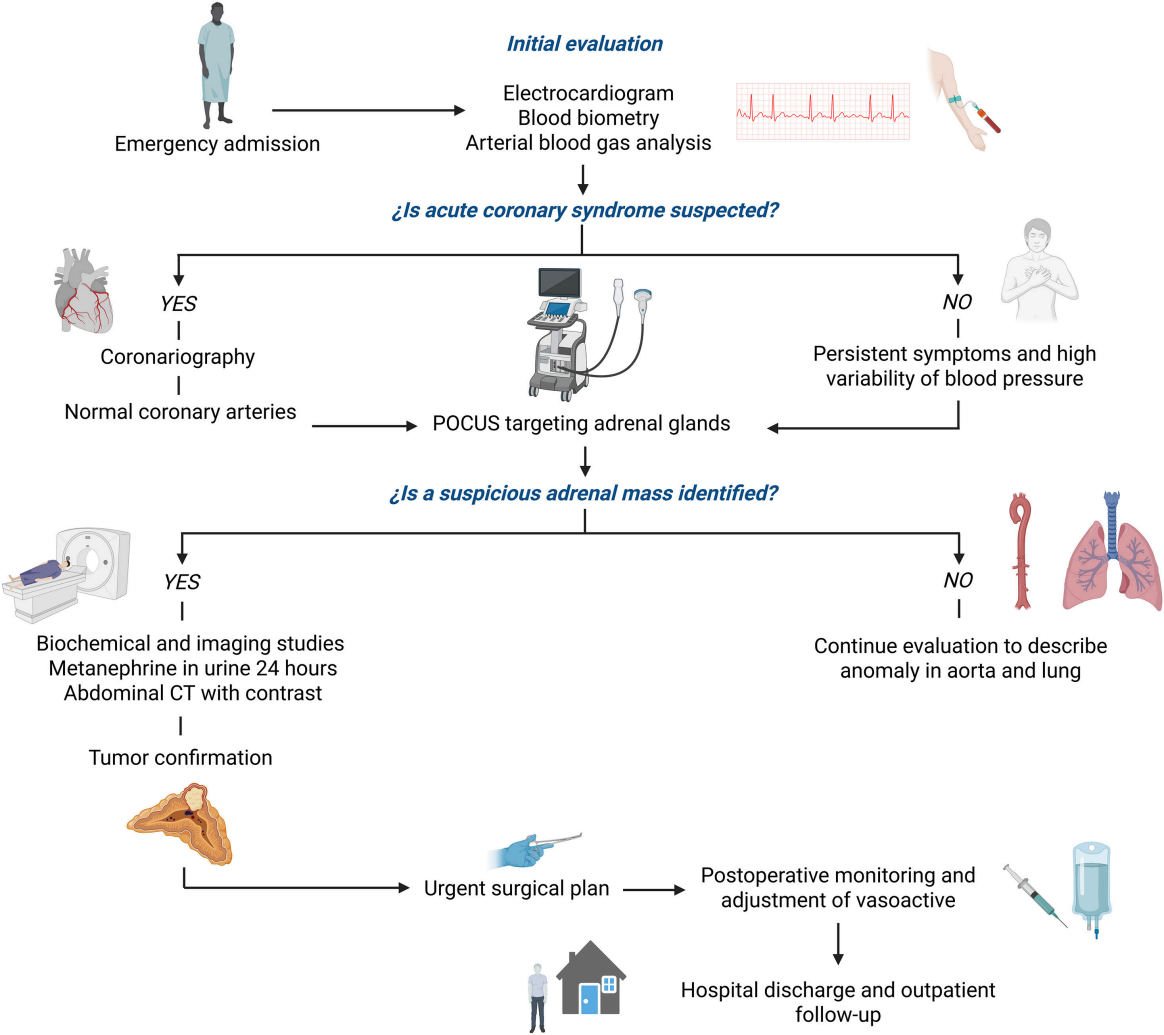
Diagnostic and therapeutic decision-making flowchart in the evaluation of a patient with suspected pheochromocytoma in an emergency setting. The chart outlines the sequential approach from initial cardiac assessment to the identification of a suprarenal mass by POCUS, guiding urgent adrenalectomy in a resource-constrained hospital in Colombia.

Adrenal vein sampling, although sometimes employed in indeterminate cases to confirm lateralization or distinguish between bilateral adrenal lesions, was not feasible in this case due to the patient’s critical hemodynamic instability. In emergent scenarios–particularly when imaging findings and clinical context strongly support the presence of a unilateral functional adrenal tumor–AVS may be omitted in favor of prompt surgical intervention. However, in hemodynamically stable patients, especially those over the age of 40, AVS can be a valuable diagnostic tool, particularly when imaging and biochemical results are inconclusive or when an incidentaloma cannot be excluded with confidence. Our clinical decision was based on the need to minimize life-threatening delays and was consistent with similar cases described in the literature. The patient experienced prolonged hemodynamic crises with cyclic patterns lasting approximately 13–16 min, posing a significant risk to his life and necessitating urgent diagnostic evaluation with POCUS. Once a lesion was identified on ultrasound and confirmed by computed tomography, adrenalectomy was determined to be the most appropriate therapeutic intervention, given the heightened risk of stroke due to hypertensive crises in the absence of metabolic or arrhythmic abnormalities. Coexisting hypotension is a known risk factor for increased perioperative morbidity and mortality, largely due to baroreceptor and autonomic dysfunction, which further emphasized the urgency of surgical intervention (20). Figure 4 presents a flowchart summarizing the clinical pathway and adaptive decision-making strategy used in this case, from initial evaluation to surgical management, particularly under resource-limited conditions.

Initially, thoracic aortic angiotomography was prioritized due to clinical suspicion of aortic dissection, based on interlimb blood pressure discrepancies and chest pain. Although a full-body CT angiogram could have identified the adrenal mass earlier, resource constraints and the immediate need to exclude life-threatening thoracic aortic pathology influenced the diagnostic strategy (21). In retrospect, a more comprehensive imaging approach might have facilitated earlier detection of the adrenal lesion and represents a missed opportunity for prompt diagnosis.

Diagnosing pheochromocytoma in the emergency department is challenging due to its non-specific presentation and the time required for confirmatory laboratory testing. In this context, POCUS emerges as a valuable tool for rapid, bedside detection (22). Although ultrasonographic characteristics specific to neuroendocrine tumors like pheochromocytoma are not well-established, direct visualization of adrenal abnormalities can significantly alter clinical decision-making, especially in scenarios marked by diagnostic ambiguity and time constraints (23, 24). Delays in surgical intervention have been linked to recurrent hypertensive crises and end-organ damage, which can be particularly detrimental in medically fragile patients (17).

While preoperative alpha-adrenergic blockade is widely recommended in pheochromocytoma management, it was omitted in this case due to the patient’s severe hemodynamic instability and risk of rapid clinical deterioration. With family consent, the surgical team proceeded with urgent adrenalectomy under intensive hemodynamic monitoring. This decision, while a deviation from established guidelines, was made after a thorough risk-benefit analysis and resulted in a favorable postoperative course without major complications. This case underscores that flexible, context-driven application of guidelines may be warranted in acute, life-threatening scenarios.

Although computed tomography remains the gold standard for adrenal imaging, POCUS cannot fully replace CT in diagnosing pheochromocytoma. Instead, it should serve as a complementary tool to help differentiate adrenal masses, such as adenomas, adrenocortical carcinomas, and metastases (25, 26). Nonetheless, CT scans may not always be readily available–especially in low-resource settings–highlighting POCUS as a radiation-free, cost-effective, repeatable, and accessible alternative for initial evaluation, particularly in emergency and outpatient settings in developing countries (27).

Surgical resection remains the definitive treatment for pheochromocytoma, though it carries substantial risks due to dramatic perioperative hemodynamic fluctuations. After tumor excision, a sudden reduction in circulating catecholamines can precipitate severe hypotension, and 10%–15% of patients may develop hypoglycemia (28–30). Consequently, vasodilators should be promptly discontinued postoperatively, and intravenous fluids administered proactively, as was done in this case (14, 31). In many instances, patients may require vasoactive agents–such as phenylephrine, norepinephrine, epinephrine, or vasopressin–to maintain blood pressure following surgery (28, 29). Although this patient required brief vasopressor support, he did not experience significant hypotension or hypoglycemia, likely reflecting the benefits of timely diagnosis and appropriate surgical management (32).

This case not only illustrates the potential regulatory and counter-regulatory effects of catecholamine-producing tumors on hemodynamics but also highlights the clinical value of POCUS, especially in emergency departments within resource-constrained settings. Its use can expedite diagnostic pathways and support timely therapeutic decisions, ultimately improving patient outcomes (33).

## 4 Patient perspective

As the patient’s condition progressed, marked by sudden and extreme fluctuations in blood pressure, his family played an increasingly central role in clinical decision-making. The initial shock of the unexpected diagnosis, combined with the urgency and complexity of the situation, was understandably overwhelming. Nonetheless, the family expressed deep appreciation for the medical team’s transparent and compassionate communication, as well as the swift and coordinated multidisciplinary response. They specifically valued the proactive use of bedside ultrasound to aid in diagnosis when conventional imaging was delayed or unavailable. The decision to proceed with emergent surgery, though difficult, was understood and supported as a life-preserving measure under exceptional circumstances. Following the procedure, the family reported a profound sense of emotional relief and expressed satisfaction with both the patient’s stable recovery and the continuity of care during follow-up.

## 5 Conclusion

Pheochromocytoma remains a challenging diagnosis, particularly in emergency department settings where its protean manifestations often mimic more common cardiovascular conditions. This case highlights how POCUS can serve as a rapid, accessible, and decisive tool in the early identification of adrenal masses, especially in resource-limited environments where advanced imaging is not immediately available. This underscores the vital role of POCUS in enhancing diagnostic accuracy and expediting treatment in critical care scenarios. The integration of POCUS into the diagnostic workflow facilitated timely surgical intervention, ultimately stabilizing the patient’s hemodynamic status and improving clinical outcomes. This experience underscores the importance of adaptable diagnostic strategies in acute care and supports the broader implementation of POCUS in emergency medicine, particularly within low- and middle-income countries.

## Data Availability

The data analyzed in this study is subject to the following licenses/restrictions: Additional case data may be requested from the corresponding author. Requests to access these datasets should be directed to juan1izquierdo11@gmail.com.
